# Examination of the Life Habits of Preschool Children Based on Their Screen Use Patterns

**DOI:** 10.3390/children11070856

**Published:** 2024-07-14

**Authors:** Tuğba Yazıcı Çakıroğlu, Özkan Sapsağlam

**Affiliations:** 1 Department of Child Care and Youth Services, Child Development Program, 29100 Gumushane, Turkey; 2 Department of Preschool Education, Yildiz Technical University, 34220 Istanbul, Turkey; ozkan@yildiz.edu.tr

**Keywords:** early childhood, screen use, life habits, eating behavior, self-care

## Abstract

Children who open their eyes to a digital world begin interacting with screens in the early years of life. The interaction between screens and children starts from the very first moments of life and intensifies over time. The aim of this study is to examine the effects of screen use patterns on the life habits of preschool children. In this context, the relationships between problematic media use, eating habits, and self-care skills among preschool children are analyzed from various perspectives. Structured according to a mixed-methods research approach, this study employs both qualitative and quantitative research designs. The study group consists of 582 children continuing their education in preschool institutions across seven different regions and nine different provinces in Turkey, along with 20 of their parents. Random and non-random sampling methods were used to form the study group. Quantitative data were collected using the Problematic Media Use Measure, Children’s Eating Behavior Inventory, and Preschool Children (36–72 months) Self-Care Skills Scale—Teacher Form, while qualitative data were gathered through a semi-structured parent interview form. The study results indicate that problematic media use and eating behaviors significantly vary according to the screen time of children, with an increase in problematic media use linked to a rise in negative eating behaviors. Parents are found to perceive the use of media devices during mealtime as a necessity, thus employing them, and believe that their children’s social behaviors are shaped according to screen content.

## 1. Introduction

The early years of life are highly influential in the development of intelligence and personality, as well as in the acquisition of social behaviors and eating habits [1]. The early childhood period, covering from birth to eight years old [2], is a time when children encounter many concepts for the first time and acquire the knowledge, skills, and behaviors they will need in later life [3]. During this period, fundamental habits are formed, and altering these habits in later years can be challenging [4].

The habits acquired by children during the preschool period through experiences have an impact on their entire lives. Self-care skills are one of the most fundamental life habits and support the development of responsibility skills [5,6]. A child’s ability to pay attention to their health and communicate and adapt to their environment and their awareness of the importance of cleanliness and nutrition are linked to social–emotional and cognitive development. In fact, supporting a child in all areas of development is very crucial and necessary for acquiring self-care skills [7]. Along with skills such as eating, toileting, and dressing and undressing, often the first to be associated with self-care, protecting oneself from accidents and dangers and recognizing safe people are also important self-care skills that need to be acquired in early childhood [8]. Within the scope of the research, eating habits and self-care skills, which are among living habits, were examined.

Nutritional habits during childhood years are one of the most decisive factors in human health [9]. Like other habits, nutritional habits are also formed during early childhood [10]. Nutritional habits affect an individual’s overall health. Unhealthy dietary practices such as consuming low-nutrient foods and skipping meals lead to various health problems and nutritional deficiencies [11]. The acquisition of nutritional and other life habits in children is influenced by computer games [12]; television [13]; friends; and other environmental factors [14].

Technological tools and applications are extremely important among the environmental stimuli that affect human life [15]. In today’s world, smartphones, online applications, and tablets are found in almost every home and have become a significant part of the daily lives of children in early childhood [16]. Preschool children typically engage with these devices to watch videos or cartoons, play games, and access mobile applications, but they also utilize them during car rides, at restaurants, and while eating. The time spent on mobile devices such as television, video game devices, computers, smartphones, and tablets is defined as “screen time” [17,18]. The amount of time children spend in front of screens is continuously increasing, negatively impacting the development process of preschool children who are particularly vulnerable [4]. The excessive use of television, computers, and mobile phones by preschool children, and the video games they play, significantly reduce the time necessary for activities essential for their healthy development, such as playing, eating, and sleeping [19].

The increasing pervasiveness of technology and media also affects the daily lives of children [20]. It is believed that technology and media have numerous effects on the developmental processes, play habits, and social interactions of children [21,22]. Technological advancements tend to keep children more attached to screens, and this negatively impacts preschool-aged children, who are developmentally vulnerable [4]. The excessive use of technology during early childhood can cause delays in cognitive, linguistic, social, or emotional development [23]. Unregulated screen-centered media use in early childhood leads to sleep disorders and memory problems [24,25,26], a decline in reading abilities and mathematical achievement during adolescence [27], poor language development and attention deficits [28], and depression [29].

Spending excessive time in front of screens leads to a decrease in physical activities among children, a shift towards ready-made and unhealthy foods, an increase in the consumption of fizzy drinks, obesity [30,31], attention deficit and hyperactivity disorders [23,32], vision problems, effects of radiation, increased body temperature, headaches, and nervous system issues [31]. It is not right to call media environments and contents merely good or bad. Some activities in digital environments present “risky opportunities” for children. Media content and technology can also have positive effects on children’s habits and developmental processes. The effects of media on children vary depending on the child’s characteristics, the content of the media, the manner of its use, and the timing of usage [33,34]. The effects of digital media may vary depending on the duration and type of use. Some researchers state that digital environments can support children’s technical skill development and identity acquisition. However, this development expectation may result in inappropriate media content and excessive use, resulting in social loneliness in different ways. Increased positive effects can be achieved by parents controlling the content and duration. Applications that are appropriate to children’s developmental levels and improve hand–eye coordination can provide positive support to children. Here, the state of influence varies depending on whether the content and duration are compatible with the child’s nature.

One of the primary reasons for children’s screen addictions is online games. Playing video games leads to the high secretion of the dopamine hormone, which increases the sensation of pleasure and subsequently contributes to addiction [35,36]. Additionally, playing video games triggers the secretion of adrenaline, which also leads to addiction. This weakens the immune system, increases inflammation, raises cortisol levels and blood pressure, and results in negative outcomes such as aggression, overstimulation, hyperactivity, and insomnia [37]. Excessive exposure to digital media tools affects not only the brain but also other organs and systems in children. Alongside the increase in obesity due to inactivity and excessive junk food consumption, a lack of self-confidence also emerges [38]. It has been observed that excessive screen time is associated with cardiovascular risk factors such as increased blood pressure, disruption of the stress regulation system (increased sympathetic arousal and irregular cortisol release), lowered HDL cholesterol, and insulin resistance [39].

The literature includes various studies on the prevalence of screen use and its effects on children [20,21,22,23,24,25,26,27,28,29,30,31,32,33,34,35,36,37,38,39,40,41]. However, it appears that there is a lack of sufficient research in the literature that examines the effects of screen use on the life habits of preschool children from different variable perspectives. The scope of the current research covering all of Turkey and including data from seven different geographic regions will fill a significant gap and make substantial contributions to the relevant literature.

Within the scope of this research, answers were sought to the following questions:

1. How does problematic media use affect the eating habits of preschool children?
2. How does problematic media use affect the self-care skills of preschool children?
3. What are the views and experiences of parents regarding the effects of problematic media use on the life habits of preschool children?

## 2. Materials and Methods

Quantitative and qualitative research methods have some limitations. Searches to overcome these limitations have led to the mixed research approach. Qualitative and quantitative research designs were used together in this study, which was structured in accordance with the mixed research approach. The main idea in choosing this method is to save the research from the limitations of the qualitative and quantitative approaches and benefit from their strengths. In this way, answers to the research questions were sought with different data collection methods and different data collection tools. The quantitative data for the study were collected using the Problematic Media Use Measure, the Children’s Eating Behavior Inventory, and the Preschool Children (36–72 months) Self-Care Skills Scale—Teacher Form. For gathering qualitative data, semi-structured interview forms conducted with parents were used.

The mixed-methods approach is a research strategy that involves collecting both quantitative data (closed-ended) and qualitative data (open-ended) to understand research problems, integrating the two datasets to reach conclusions. The fundamental assumption of this approach is that combining statistical tendencies (quantitative data) with narratives and personal experiences (qualitative data) provides more advantages than using either method alone in understanding the research problem [42].

This study used a correlational (relational) research design as the quantitative research pattern. In the correlational research model, the relationships or effects between variables are determined through a correlation coefficient [43]. The correlational research model can be conducted using two types of methods: correlation-type relationships and comparisons. In correlational research, it is determined whether variables change together, and if so, how this change occurs. This approach helps to identify attitudes and trends [44].

The qualitative part of the research adopted the case study design, which focuses on the detailed examination of one or more instances within a confined system [45]. There are three different types of case studies: explanatory case studies, exploratory case studies, and descriptive case studies [46]. Merriam [47] notes that a case study allows for the detailed description and examination of an existing system in a limited way. In this research, the specific case being examined is the impact of problematic media use on children’s life habits.

### 2.1. Measurement Tools

The quantitative research data of the study were obtained through the Problematic Media Use Measure [48], the Children’s Eating Behavior Inventory [49], and the Self-Care Skills Scale for Preschool Children (36–72 months)—Teacher Form [50]. The qualitative data were collected via a semi-structured parent interview form developed by the researchers.

Problematic Media Use Measure (PMUM): This scale has been developed to detect problematic media use in children aged 4–11. The scale has been adapted into Turkish by Furuncu and Öztürk [48]. The scale is a 5-point Likert type scale consisting of 9 items and one sub-dimension. The Cronbach’s alpha value of the scale is calculated as 0.90.

Children’s Eating Behavior Inventory: This scale has been developed to determine the eating behaviors of children aged 2–12. It has been adapted into Turkish by Ünlü [49]. The scale consists of two sub-dimensions and 40 items. The Cronbach’s alpha value for the first sub-dimension, “The Child’s Positive Eating Behaviors”, is 0.79, and the Cronbach’s alpha value for the second sub-dimension, “Negative Eating Behaviors Shown by Child”, is 0.68.

Preschool Children (36–72 months) Self-Care Skills Scale—Teacher Form: This was developed by Dinçer, Demiriz, and Ergül [50] to determine the self-care skills of children attending preschool institutions. The scale is a 4-point Likert type scale consisting of 46 items and four sub-dimensions. The Cronbach’s alpha value of the sub-dimensions are as follows: dressing–undressing, 0.93; order, 0.89; eating, 0.86; personal cleaning—toilet, 0.79. The Cronbach’s alpha value of the scale was calculated as 0.95.

Parent Interview Form: This was prepared by the researchers after reviewing the relevant literature. It includes open-ended questions answered by parents, aimed at determining children’s media use preferences and the effects of media on children. The interview form includes 10 questions about parents’ opinions and experiences regarding the effects of media on children’s life habits.

### 2.2. Participants

The study group consisted of 582 children aged 36–69 months attending preschools affiliated with the Ministry of National Education in nine different provinces across seven regions of Turkey, as well as 20 parents. In forming the study group, simple random sampling was used for the quantitative part of the research, and homogeneous sampling was used for the qualitative part. Simple random sampling involves the random selection of sampling units from the population list, ensuring that all units have an equal chance of being selected. Homogeneous sampling involves selecting a single subgroup with similar characteristics from the population related to the research problem [51]. At the beginning of this study, the aim was for the participants to be homogeneous. However, differences in Turkish family culture have caused the mother to come to the fore in caring for the child. Mothers generally give feedback in the child’s education during the preschool period. Preschool teachers who provide education to the child generally communicate with the mother first, as she is typically always available. Mothers are primarily involved in most of the stages when the child returns from school and prepares for the next day (including homework given by school and forms requested by the teacher). The demographic information of the participants in the study group is presented in Table 1.

The first study was conducted with a sample of 582 children, comprising 301 males (52%) and 281 females (48%). The distribution of children’s screen use time was as follows: 0–30 min (39 children, 7%), 31–60 min (77 children, 13%), 1 h or more (136 children, 23%), 2 h or more (178 children, 31%), 3 h or more (104 children, 18%), and 4 h or more (48 children, 8%).

The second study involved 20 parents, with 1 male (5%) and 19 females (95%). The reported screen use time for their children was as follows: 0–30 min (3 children, 15%), 31–60 min (4 children, 20%), 1 h or more (4 children, 20%), 2 h or more (4 children, 20%), 3 h or more (3 children, 15%), and 4 h or more (2 children, 10%). The majority of our sample consisted of mothers. Since we have a culturally patriarchal family structure, in most of our families, the father is the parent who works outside the home, earns money, and does jobs that require more muscle power. Even though the mother works outside, she is the primary parent who takes care of the children’s care and education and interacts with people in the education process. It is stated that pre-school teachers in Turkey are predominantly women. This is primarily a factor in female teachers’ communication with mothers. It is thought that another reason for this situation is that life habits and self-care skills are imparted to children mostly through mothers.

### 2.3. Data Collection and Analysis

Ethics committee permission was first obtained during the research process. The study protocol has been approved by Yildiz Technical University unit of Scientific Research and Ethical Review Board (Report Number: h28.02.2022/2022.02). The study was performed in accordance with the ethical standards laid down in the 1964 Declaration of Helsinki and its following updates. The voluntary participation of all participants was sought, and voluntary participation consent was obtained from the participants.

The research data were collected by the researchers from 582 children aged 36–69 months and 20 parents in nine different provinces across seven regions of Turkey, at preschools affiliated with the Ministry of National Education. During the data collection process, permission was obtained from the Academic Ethics Committee of the university where the researchers work and from the Strategy Development Department of the Turkish Ministry of National Education. Participants were informed that participation in the research was voluntary. They were assured that no questions beyond the scope of the research would be asked, there would be no intrusion into their privacy, their personal rights and confidentiality would not be violated, and their personal information would not be used. The researchers provided information to the participants about the measurement tools and scoring. Afterward, the measurement tools were administered, and the forms were collected. The data collection process took approximately six months. The “Problematic Media Use Measure” and the “Children’s Eating Behavior Inventory” were filled out by 582 parents, considering the relevant characteristics of their own children. The “Preschool Children (36–72 months) Self-Care Skills Scale—Teacher Form” was completed by 65 preschool teachers, considering the same 582 children. The “Parent Interview Form” was administered to 20 parents through face-to-face interviews conducted by the researchers. Due to incorrect and incomplete markings, the scales filled out by 82 participants were excluded from the evaluation.

During the analysis process of quantitative data, outliers were initially examined, and the skewness and kurtosis values of the variables were checked. To meet the conditions of normality, outliers were examined, and 82 data points were removed from the dataset. As seen in Table 2, after removing these data points, the range of values obtained by dividing the skewness and kurtosis values by the standard error was between −0.545 and 2.653. For normal distribution, the values obtained by dividing the skewness and kurtosis values by the standard error should be between +3 and −3 [52]. In this case, since the research data met the conditions for normal distribution, the data were analyzed using parametric methods. The skewness and kurtosis values are presented in Table 2.

Validity and reliability in scientific research are crucial for the acceptability of research findings. Different methods and techniques must be used to ensure validity and reliability in qualitative research. In this study, the triangulation technique, one of the validity and reliability methods suggested by Patton, was used. There are four types of triangulation techniques: method triangulation, data source triangulation, analyst triangulation, and theory/perspective triangulation [53]. To determine the validity and reliability of the qualitative data analysis results, data source triangulation (using both qualitative and quantitative data) and analyst triangulation (the independent analysis of research data by researchers) were employed. During the analysis of research data, themes and codes were independently created by researchers for the responses to the interview questions directed at parents. Coding is the process of identifying different aspects of data and marking or labeling them in small segments [42,47,54,55]. The themes and codes created were independently coded by researchers, and consistency between coders was determined. The reliability of the data analysis was determined using the formula by Miles and Huberman [55]: Agreement Percentage = [Agreement/(Agreement + Disagreement) × 100]. Accordingly, the agreement percentage between coders for the interview responses was found to be 94%.

## 3. Results

### 3.1. Quantitative Results

The relationships between variables have been thoroughly examined. Both qualitative and quantitative findings have been analyzed and summarized separately.

A one-way analysis of variance (ANOVA) was conducted to determine whether the participants’ mean scores on the study variables differed among the screen time use groups. The results are presented in the tables above. As shown in Table 3, the one-way ANOVA conducted to determine whether there was a significant difference in problematic media use scores based on the “screen time use” variable indicated that there was no statistically significant difference between the groups [F(5,576) = 18.766; *p* < 0.05]. Tukey’s analysis was applied to determine between which groups the differences occurred. According to the analysis results, the groups where the differences occurred were identified. The differences were found between the following groups: 0–30 min ($\bar{X}$ = 13.98) and 3 h ($\bar{X}$ = 18.11), 0–30 min ($\bar{X}$ = 13.98) and 4 h or more ($\bar{X}$ = 23.79), 30–60 min ($\bar{X}$ = 13.38) and 1 h ($\bar{X}$ = 16.36), 30–60 min ($\bar{X}$ = 13.38) and 4 h or more ($\bar{X}$ = 23.79), 1 h ($\bar{X}$ = 16.36) and 4 h or more ($\bar{X}$ = 23.79), 2 h ($\bar{X}$ = 17.63) and 4 h or more ($\bar{X}$ = 23.79), and 3 h ($\bar{X}$ = 18.11) and 4 h or more ($\bar{X}$ = 23.79).

The Self-Care Skills scores were examined considering the “screen time use” variable, and no statistically significant difference was found among the groups [F (5,576) = 0.242; *p* > 0.05]. Similarly, the score of The Child’s Positive Eating Behaviors were analyzed based on the same variable, and no statistically significant difference was detected among the groups [F (5,576) = 0.738; *p* > 0.05].

On the other hand, the Negative Eating Behaviors Shown by Child During the Meal scores of the child were evaluated according to “screen time use”, and a significant difference was observed among the groups [F (5,576) = 2.524; *p* < 0.05]. In this sub-dimension, the one-way ANOVA conducted for the Negative Eating Behaviors Shown by Child After the Mealtime scores of the child revealed a statistically significant difference among the groups [F (5,576) = 7.572; *p* < 0.05]. The sources of the differences were determined through Tukey’s analysis, showing that the differences were between the following groups: 0–30 min ($\bar{X}$ = 12.31) and 4 h or more ($\bar{X}$ = 15.77), 30–60 min ($\bar{X}$ = 12.31) and 1 h ($\bar{X}$ = 12.67), 30–60 min ($\bar{X}$ = 12.31) and 4 h or more ($\bar{X}$ = 15.77), 1 h ($\bar{X}$ = 12.067) and 2 h ($\bar{X}$ = 12.85), 2 h ($\bar{X}$ = 12.85) and 4 h or more ($\bar{X}$ = 15.77), and 3 h ($\bar{X}$ = 13.77) and 4 h or more ($\bar{X}$ = 15.77).

Analyzing the “Opinions and Behaviors of Mother “scores in relation to “screen time use” revealed no statistically significant difference among the groups [F (5,576) = 1.241; *p* > 0.05]. Additionally, the Child’s Behavior Related to Food Preparation scores were evaluated based on the same variable, and a statistically significant difference was detected among the groups [F (5,576) = 2.807; *p* < 0.05]. Tukey’s analysis identified the sources of these differences, showing that the differences were between the following groups: 0–30 min ($\bar{X}$ = 8.30) and 4 h or more ($\bar{X}$ = 6.88), 30–60 min ($\bar{X}$ = 7.90) and 4 h or more ($\bar{X}$ = 6.88), and 1 h ($\bar{X}$ = 7.94) and 4 h or more ($\bar{X}$ = 6.88).

Finally, evaluating the “Adverse Conditions in terms of Child at the Mealtime” scores from the child’s perspective in relation to “screen time use” revealed no statistically significant difference among the groups [F (5,576) = 1.569; *p* > 0.05].

The results of the Pearson correlation analysis conducted to determine the level and direction of relationships between the variables in the study are presented in Table 4. The relationships between Problematic Media Use and other variables were examined, and it was found that there were statistically significant positive correlations between Problematic Media Use and the sub-dimensions of the Children’s Eating Behavior Inventory, specifically Negative Eating Behaviors Shown by Child During the Meal (r = 0.309, *p* < 0.01), Negative Eating Behaviors Shown by Child After the Mealtime (r = 0.442, *p* < 0.01), Opinions and Behaviors of Mother (r = 0.210, *p* < 0.01), and Adverse Conditions in terms of Child at the Mealtime (r = 0.202, *p* < 0.01). Additionally, there were statistically significant negative correlations between Problematic Media Use and the Child’s Positive Eating Behaviors (r = −0.112, *p* < 0.01) and the Child’s Behavior Related to Food Preparation (r = −0.194, *p* < 0.01). No significant correlation was found in the correlation analysis between the variables of Problematic Media Use and Self-Care Skills. The responses of children to parental screen time warnings are presented in Table 5.

The proportional distribution of children’s responses when warned by their parents during their use of digital devices is presented in Table 5. The responses are coded as follows: Response 1—“Hears but continues to use” (*n* = 121), Response 2—“Cries or screams to avoid stopping” (*n* = 75), Response 3—“Stops using immediately” (*n* = 89), Response 4—“Ignores the warning” (*n* = 66), and Response 5—“Insists on using a bit longer” (*n* = 346). According to the analysis of children’s responses, the lowest proportion was for Response 4—“Ignores the warning”, and the highest proportion was for Response 5—“Insists on using a bit longer”.

### 3.2. Qualitative Findings

To gain a multifaceted and in-depth perspective of the situation, the researchers conducted face-to-face interviews with 20 parents who have children with different screen use habits. The parents’ views on children’s media usage preferences are presented in Table 6.

As shown in Table 6, the results obtained from the semi-structured interview forms applied to parents indicate that children’s media usage preferences are mainly driven by the purposes of “escaping from life tasks, non-task-related needs, imitating others, learning solutions, traditional solutions, and being influenced by heroes”. Children’s media content preferences predominantly include “online games, cartoons, YouTube videos, and toy videos”. The patterns of children’s media usage are characterized by “forgetting life needs, managing social environment, preference for non-screen games, refusal to be interrupted, and staying connected with the environment”. Some example statements from participants are provided below.

*“When he needs to use the toilet, he holds it until the last moment to avoid putting down the tablet, and only goes when he can’t hold it any longer”.*

(P1/Escaping from life tasks)

*“If the phone or tablet he is using runs out of battery or breaks, he has a crying fit and usually throws himself on the ground”.*

(P3/Refusal to be interrupted)

*“Recently, unfortunately, he won’t eat without watching a video. He always wants to watch something on the video”.*

(P6/Non-task-related needs)

*“Mostly watches cartoons or unboxing videos”.*

(P11/Cartoons, Toy videos)

*“He tries to solve problems with what he sees in the media”.*

(P18/Learning solutions)

As shown in Table 7, the results obtained from the semi-structured interview forms administered to parents indicate that the effects of screen use on children’s life skills are particularly evident in the areas of “social life, responsibility, self-awareness, decision-making, survival, and self-care”. The effects of screen use on children’s habits predominantly include “eating habits, use of devices, and play habits”. The effects on children’s communication skills mainly encompass “spoken language, behaviors, body language, and communicative response”. The behavior problems observed in children are mainly “refusing to eat, imitating heroes, objecting, and using slang words”. It was noted that parents especially expressed the necessity of using digital media tools to get their children to eat, and they believed that the behavior models their children used in social relationships were shaped by the content they were exposed to on screens. Some example statements from participants are provided below.

*“In daily conversations, he uses phrases like ‘don’t forget to like’ with his friends”.*

(P4/Behaviors)

*“He neglects even basic needs like eating just to use the screen”.*

(P7/Refusing to eat)

*“Sometimes he gets so absorbed in TV that he comes to the dinner table late”.*

(P10/Self-care)

*“When he has a responsibility that concerns him, he doesn’t respond when we call him”.*

(P16/Responsibility)

*“He imitates what he sees in media environments. For example, he says things like ‘Mom, I am a zombie’”.*

(P19/Imitating heroes)

## 4. Discussion

This study examined the effects of screen use patterns on the life habits of preschool children. According to the research results, children’s Problematic Media Use levels and some sub-dimension scores of the Children’s Eating Behavior Inventory (Negative Eating Behaviors Shown by Child During the Meal, Negative Eating Behaviors Shown by Child After the Mealtime, Child’s Behavior Related to Food Preparation) significantly differ according to the ‘screen use time’ variable. It was found that the Self-Care Skills scores and some sub-dimensions of the Children’s Eating Behavior Inventory (Opinions and Behaviors of Mother, Adverse Conditions in terms of Child at the Mealtime) do not statistically significantly differ according to the ‘screen use time’ variable.

Children who use screens for four hours or more have higher levels of Problematic Media Use, Negative Eating Behaviors Shown by Child During the Meal, and Negative Eating Behaviors Shown by Child After the Mealtime compared to other groups. As children’s screen time increases, so do problematic media use and negative eating behaviors. Research [56] shows that in Turkey, the internet usage time for children aged three to six ranges from one to four hours. Piotrowski et al. [57] found that three-year-old children spend most of their media usage time watching television, and by the age of four to six, video games are added to their daily media usage, with an average of 30 min per day. Kabali et al. [58] reported that 70% of parents allow their children to use electronic devices when focusing on their own work, 65% to calm their children, and 29% before putting them to sleep. Pembecioğlu [59] noted that although screen use in children initially appears harmless in terms of content, it can eventually lead to addiction or behavioral disorders. Tokgöz [60] found that families leave their children unsupervised while watching television, leading children to consume snacks uncontrollably and thus causing eating disorders. Kobak and Pek [61] reported that parents visiting a mother–child health clinic often feed their children in front of the television. Lepicnik Vodopivec [62] stated that parents use media tools to effectively influence and feed their children. Van den Bulck and Eggermont [63] indicated that screen use in children is associated with increased meal skipping and unhealthy eating habits. Children who watch television for 4 h or more daily are seven times more likely to skip meals weekly, and those who play computer games at least four times a week are nine times more likely to skip meals weekly.

The research results show that as screen time increases, so do problematic media use and negative eating behaviors in preschool children. The relevant literature shows that parents direct their children to use screens when they are busy and parents use electronic devices and media as a tool to feed their children. The time spent in front of the screen increases, and the time spent on other life activities decreases. Spending more time in front of a screen and problematic media usage habits are thought to negatively affect preschool children in two ways. Firstly, the time spent in front of the screen reduces the time spent on other life habits. Secondly, due to the increase in the time spent in front of the screen, the negative media content that children are exposed to increases. It can be said that the research results are consistent with the relevant literature.

The Pearson correlation analysis was conducted to determine the level and direction of relationships between the variables in the study. Various findings were obtained regarding the relationships between Problematic Media Use and other variables. Significant positive correlations were found between the sub-dimensions of the Children’s Eating Behavior Inventory, specifically Negative Eating Behaviors Shown by Child During the Meal (r = 0.309, *p* < 0.01) and Negative Eating Behaviors Shown by Child After the Mealtime (r = 0.442, *p* < 0.01). Additionally, significant positive correlations were found between Opinions and Behaviors of Mother (r = 0.210, *p* < 0.01) and Adverse Conditions in terms of Child at the Mealtime (r = 0.202, *p* < 0.01). On the other hand, significant negative correlations were found between the Child’s Positive Eating Behaviors (r = −0.112, *p* < 0.01) and the Child’s Behavior Related to Food Preparation (r = −0.194, *p* < 0.01). No significant correlation was found in the correlation analysis between the Problematic Media Use and Self-Care Skills variables.

As Problematic Media Use increases, scores for the sub-dimensions of the Children’s Eating Behavior Inventory, such as Negative Eating Behaviors Shown by Child During the Meal, Negative Eating Behaviors Shown by Child After the Mealtime, Opinions and Behaviors of Mother, and Adverse Conditions in terms of Child at the Mealtime, also increase. The world we live in today is defined by concepts such as “digital worlds”, “virtual universes”, and “electronic villages”. Media changes our life habits, preferences, relationships, attitudes, and behaviors. Preschoolers are the ones most defenseless against the possible harms of media content, because preschool children do not yet have a perception of reality. That is why they think many things they see in advertisements and other media are real. Zimmerman and Bell [64] state that unhealthy food advertisements promote consumption and pose a health risk. Montgomery and Chester [65] note that children’s exposure to advertising influences their food choices. Harris et al. [66] report that children’s nutrient intake increases by 45% when they are exposed to advertising during screen time. Another study found that children aged 6–84 months who watch media devices while eating have screen exposure times of 225 min, compared to 140 min for those who do not [67]. Research conducted by Jusiene and colleagues [68] with 847 children between the ages of two and five has determined that screen use during meals is associated with daily screen time, snack consumption, and emotional and behavioral problems in children. Wenhold and Harrison [69] report that screen use during meals reduces the quality of communication among family members. A study on media use among preschool children by Gündoğdu and colleagues [70] has determined that 86% of parents most frequently use media devices during meal times. The research findings, in line with the literature mentioned above, show that problematic media use leads to an increase in children’s negative eating behaviors. It can be said that the research results are consistent with the related literature.

Another significant result of the research is the reactions children give when warned by their parents during their use of digital media devices. The reactions are coded as follows: Response 1—Hears but continues to use (*n* = 121), Response 2—Cries or screams to avoid stopping (*n* = 75), Response 3—Stops using immediately (*n* = 89), Response 4—Ignores the warning (*n* = 66), and Response 5—Insists on using a bit longer (*n* = 346). According to the analysis of children’s responses, the lowest proportion was Response 4—Ignores the warning (*n* = 66), and the highest proportion was Response 5—Insists on using a bit longer (*n* = 346). Research findings show that children have difficulty leaving the screen and resist their parents in order to not to leave the screen. One of the negative effects of increasing screen usage time for children is that children become uninterested in non-screen activities. Children who cannot find as much speed, flow, excitement, and pleasure in the real world as in the virtual universe do not want to leave screens and media environments. However, children need real-life experiences to learn life skills. New types of addiction have emerged due to the development of technology and the spread of media. According to the American Psychiatric Association, in the Diagnostic and Statistical Manual of Mental Disorders-5 (DSM-5), the internet gaming disorder category is included. Some research results, examples of which are given below, also show that media and screen use is becoming increasingly common among children.

Media use reports indicate that children aged 3 to 6 spend approximately three hours in front of screens [17]. Sapsağlam [22] notes that even children as young as 3 years old are aware of social media applications. It is reported that more than half of children under the age of eight regularly have access to mobile devices at home [17]. According to the research by Ateş and Durmuşoğlu Saltalı [71], about half of the parents expressed that they could not control their children’s use of tablets and mobile phones, while the majority stated that they could control the content. This important and meaningful result aligns with the literature, showing that as children’s screen time and interactions with media increase, they find it more challenging to detach from screens, which also poses challenges for parents.

When the results of the semi-structured interviews conducted with the parents were examined, it has been observed that they think that their children are influenced by characters in media environments and that the most affected life habits are eating habits, using objects, and playing habits. It has also been observed that parents think that their children’s language and speaking habits and communication skills in general are negatively affected as a result of their exposure to media content. Parents stated that the most important behavioral problems caused by the media are not eating, imitating negative media characters, and using slang.

As children’s screen usage time increases, their level of influence from positive and negative media content also increases. Aral and Doğankeskin [72] state that children aged 0–6 use mobile phones (44.8%), tablets (43.1%), computers (21%), and televisions (70.2%) primarily for “watching cartoons and playing digital games”. Toksoy [73] notes that children are exposed to more screen use than in previous years and frequently follow YouTube broadcasters. Children who realize that they are individuals want to define and complete their identities. In this process, they look for role models for themselves. Media offers children many positive and negative role models. Ergüney [56] indicates that children tend to identify with the characters they watch in cartoons and videos, wanting to be beautiful, dress, eat, and speak like those characters. Ertürk [74] states that media tools enable children to socialize, learn, and develop and have an environmental impact, and that the messages conveyed by media tools result in changes in behavior, emotions, and thoughts. The study by Akıncı and Orhan [13] reveals that children eating while watching television leads to unhealthy eating habits and increases the tendency towards a sedentary lifestyle. Akyar and Sapsağlam [75] state that media content and screen use affect children’s daily life habits.

One of the important findings of the research is the views of parents that media content and screen usage time change children’s communication styles. In particular, the speech of children who follow YouTubers is faster, more interrupted, and mostly contains technology content. Toksoy [73] noted that preschool children remember phrases used by people in the videos they watch, such as “Hello/welcome/greetings” and “Subscribe/Don’t forget to subscribe to my channel”. They also hear slang and curse words, as well as phrases like “Hit”, “Followers”, and “Challenge” from these videos. Significant changes have been observed in the speech patterns and behaviors of children, including altering their voices when speaking, speaking with a stronger tone, and producing more intense sounds like “aaa, waaaa”.

The research results indicate that the increase in children’s screen time and their tendency towards problematic media use negatively affect their life habits. The quantitative and qualitative findings of the research consistently support this outcome. Based on the research results, the following recommendations are made:

- It is recommended to examine the research problem from an intercultural perspective.
- It is recommended that parents and teachers take more responsibility to ensure that children have fun and learn through real-life experiences that do not involve screens.
- The impact of parents’ screen use times and media usage habits on children’s screen and media usage habits should be examined.
- The mediating roles of parents in children’s screen and media usage should be studied.
- Media platforms and content that support children’s life habits, such as eating and self-care, should be developed and promoted.
- Media literacy training should be organized for children, parents, and teachers, and their skills in this area should be supported.
- It is recommended that studies in the field of digital media, child health, and welfare should be approached from an interdisciplinary perspective and research in this field should be increased.

This research has some limitations that should be acknowledged. The sample only includes children within a specific age range, which limits the generalizability of the findings. The screen use habits of children in different age groups and their effects on lifestyle may differ significantly. Additionally, life habits may vary with cultural differences; the research was only conducted with participants from Turkey. The results were obtained with samples selected from seven existing regions of Turkey. Turkey’s eastern, western, northern, southern, and central regions show local cultural differences. For example, according to the results of the self-care scale we applied, the children of the families participating in the survey in the eastern region were seen as more competent in performing many skills. It is thought that the reason for this is that children in the east are left to struggle with life alone from an early age. Since families’ income sources are generally agriculture and animal husbandry, children in this region begin to help the family earn their living at an earlier age. Therefore, the rate of having skills is increased compared to their peers in other regions. This study may not fully reflect such variations.

**Figure 1:**
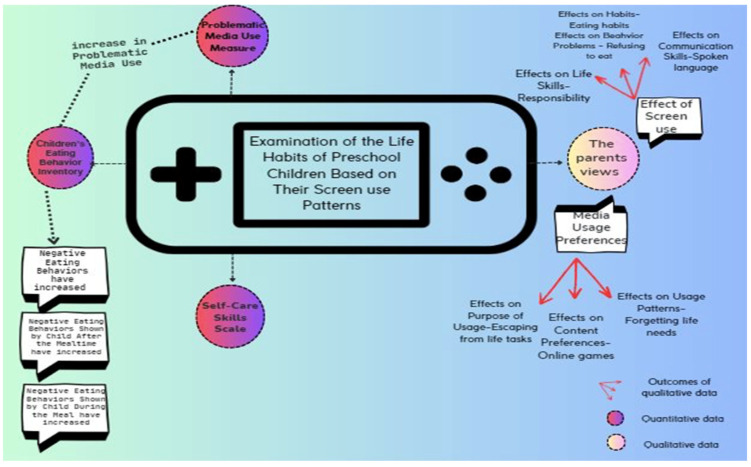
The research process, method, and important results.

The research process, method, and important results are summarized in Figure 1.

## Figures and Tables

**Table 1:** Information of participants.

Study	Variables		f	%
Study I (Quantitative Research)	Parent’s Gender	Male	92	16
		Female	490	84
	Child’s Gender	Male	301	52
		Female	281	48
	Child’s Screen Use Time	0–30 min	39	7
		31–60 min	77	13
		1 h or More	136	23
		2 h or More	178	31
		3 h or More	104	18
		4 h or More	48	8
Study II (Qualitative Research)	Parent’s Gender	Male	1	5
		Female	19	95
	Child’s Gender	Male	11	55
		Female	9	45
	Child’s Screen Use Time	0–30 min	3	15
		31–60 min	4	20
		1 h or More	4	20
		2 h or More	4	20
		3 h or More	3	15
		4 h or More	2	10

**Table 2:** Skewness and kurtosis values.

Variables	Skewness	Kurtosis
Problematic Media Use	0.1130	1384
Self-care Skills	−0.165	−0.370
The Child’s Positive Eating Behaviors	−0.545	0.059
Negative Eating Behaviors Shown by Child During the Meal	0.716	0.505
Negative Eating Behaviors Shown by Child After the Mealtime	0.752	0.755
Opinions and Behaviors of Mother	0.261	0.666
Child’s Behavior Related to Food Preparation	0.366	0.133
Adverse Conditions in terms of Child at the Mealtime	1.771	2.653

**Table 3:** An examination of lifestyle habits based on screen time.

Variables		SS	df	MS	F	*p*
Problematic Media Use	Between Group	3,820,578	5	764,116	18,766	0.000 *
	Within Group	23,453,005	576	40,717		
	Total	27,273,584	581			
Self-care Skills	Between Group	477,632	5	95,526	0.242	0.944
	Within Group	226,976,290	576	394,056		
	Total	227,453,923	581			
The Child’s Positive Eating Behaviors	Between Group	39,780	5	7.956	0.738	0.596
	Within Group	6,213,349	576	10,787		
	Total	6,253,129	581			
Negative Eating Behaviors Shown by Child During the Meal	Between Group	151,938	5	30,388	2.524	0.028 *
	Within Group	6,933,974	576	12,038		
	Total	7,085,913	581			
Negative Eating Behaviors Shown by Child After the Mealtime	Between Group	496,510	5	99,302	7.572	0.000 *
	Within Group	7,554,325	576	13,115		
	Total	8,050,835	581			
Opinions and Behaviors of Mother	Between Group	43,132	5	8.626	1.241	0.288
	Within Group	4,003,428	576	6.950		
	Total	4,046,559	581			
Child’s Behavior Related to Food Preparation	Between Group	58,601	5	11,720	2.807	0.016 *
	Within Group	2,405,051	576	4.175		
	Total	2,463,652	581			
Adverse Conditions in terms of Child at the Mealtime	Between Group	7.287	5	1.457	1.569	0.167
	Within Group	534,985	576	0.929		
	Total	542,272	581			

* *p* < 0.05.

**Table 4:** Pearson correlation of the relationships between problematic media use and eating behaviors.

Variables	1	2	3	4	5	6	7
1. Problematic Media Use	1						
2. Positive Eating Behaviors of the Child	−0.112 **	1					
3. Negative Eating Behaviors of the Child During Meals	0.309 **	−0.322 **	1				
4. Negative Eating Behaviors of the Child Outside of Meal Times	0.442 **	0.048	0.264 **	1			
5. Mothers’ Perspectives and Behaviors	0.210 **	−0.077 *	0.420 **	0.206 **	1		
6. Child’s Behaviors Regarding Meal Preparation	−0.194 **	0.222 **	−0.011	0.027	0.001	1	
7. Negative Situations Arising for the Child During Meals	0.202 **	−0.226**	0.379 **	0.220 **	0.208 **	0.096 *	1

* *p* < 0.05; ** *p* < 0.01.

**Table 5:** Responses of children to parental screen time warnings.

Response	*n*	%
Response 1	121	20.8
Response 2	75	12.9
Response 3	89	32.5
Response 4	66	11.3
Response 5	346	59.5

**Table 6:** Parents’ views on children’s screen use habits.

Theme	Sub-Theme	Codes
Media Usage Preferences	Purpose of Usage	Escaping from life tasks Non-task-related needs İmitating others Learning solutions
	Content Preferences	Online games Cartoons YouTube videos Toy videos
	Usage Patterns	Forgetting life needs Managing social environment Preference for non-screen games Refusal to be interrupted Staying connected with the environment

**Table 7:** Parents’ views on the effects of screen use on children.

Theme	Sub-Theme	Codes
Effect of Screen use	Effects on Life Skills	Social life Responsibility Self-awareness Decision-making Survival Self-care
	Effects on Habits	Eating habits Use of devices Play habits
	Effects on Communication Skills	Spoken language Behaviors Body language Communicative response
	Behavioral Problems	Refusing to eat Imitating heroes Objecting Using slang words

## Data Availability

The data presented in this study could be available on request from the corresponding author.
